# Infection prevention and control for COVID-19 response in the Rohingya refugee camps in Bangladesh: an intra-action review

**DOI:** 10.1186/s12939-023-01926-2

**Published:** 2023-06-06

**Authors:** Rebecca Racheal Apolot, Simon Ssentamu Kaddu, Egmond Samir Evers, Paul Debashish, S. M. Niaz Mowla, Sabbir Ahmed, Aritra Das, Abu Toha M. R. H. Bhuiyan, Md Mahbubur Rahman, Anupam Barua, Allen Gidraf Kahindo Maina, Murad Sultan, Marsela Nyawara, Victoria Willet, Kai Von Harbou

**Affiliations:** 1 World Health Organization, Cox’s Bazar Emergency Sub Office, Cox’s Bazar, Bangladesh; 2 Food for the Hungry/Medical Teams International, Cox’s Bazar, Bangladesh; 3 Office of the Refugee Relief and Repatriation Commissioner, Cox’s Bazar, Bangladesh; 4 Civil Surgeon’s Office, Cox’s Bazar, Bangladesh; 5 Cox’s Bazar Medical College, Cox’s Bazar, Bangladesh; 6 United Nations High Commissioner for Refugees, Cox’s Bazar Sub Office, Cox’s Bazar, Bangladesh; 7 World Health Organization, Bangladesh Country Office, Dhaka, Bangladesh; 8 International Organization for Migration (IOM), Cox’s Bazar Sub Office, Cox’s Bazar, Bangladesh; 9 World Health Organization, WHO Health Emergencies (WHE) Programme, Geneva, Switzerland

**Keywords:** Infection Prevention and Control, COVID-19, Rohingya, Refugees, Lessons learned, Bangladesh, Intra-action review

## Abstract

**Background:**

Infection Prevention and Control (IPC) is critical in controlling the COVID-19 pandemic and is one of the pillars of the WHO COVID-19 Strategic Preparedness and Response Plan 2020. We conducted an Intra-Action Review (IAR) of IPC response efforts to the COVID-19 pandemic in Cox's Bazar, Bangladesh, to identify best practices, challenges, and recommendations for improvement of the current and future responses.

**Methods:**

We conducted two meetings with 54 participants purposively selected from different organizations and agencies involved in the frontline implementation of IPC in Cox's Bazar district, Bangladesh. We used the IPC trigger questions from the WHO country COVID-19 IAR: trigger question database to guide the discussions. Meeting notes and transcripts were then analyzed manually using content analysis, and results were presented in text and quotes.

**Results:**

Best practices included: assessments, a response plan, a working group, trainings, early case identification and isolation, hand hygiene in Health Facilities (HFs), monitoring and feedback, general masking in HFs, supportive supervision, design, infrastructure and environmental controls in Severe Acute Respiratory Infection Isolation and Treatment Centers (SARI ITCs) and HFs and waste management. Challenges included: frequent breakdown of incinerators, limited PPE supply, inconsistent adherence to IPC, lack of availability of uniforms for health workers, in particular cultural and gender appropriate uniforms and Personal Protective Equipment (PPE). Recommendations from the IAR were: (1) to promote the institutionalization of IPC, programs in HFs (2) establishment of IPC monitoring mechanisms in all HCFs, (3) strengthening IPC education and training in health care facilities, and (4) strengthen public health and social measures in communities.

**Conclusion:**

Establishing IPC programmes that include monitoring and continuous training are critical in promoting consistent and adaptive IPC practices. Response to a pandemic crisis combined with concurrent emergencies, such as protracted displacement of populations with many diverse actors, can only be successful with highly coordinated planning, leadership, resource mobilization, and close supervision.

**Supplementary Information:**

The online version contains supplementary material available at 10.1186/s12939-023-01926-2.

## Background

The World Health Organization (WHO) declared COVID-19 a pandemic on 11 March 2020 and called upon countries to scale up their COVID-19 response [1]. On 8 March 2020, Bangladesh reported its first case of COVID-19 [2], while Cox's Bazar district reported the first case on 15 April 2020 and the first case of COVID-19 was reported in the Rohingya refugee camps on 15 May 2020. This Intra-Action Review (IAR) was completed on 02 February 2021 at which time, Cox's Bazar district had reported 5505 confirmed cases of COVID-19 and 73 deaths. Specifically, for Rohingya refugees, 381 confirmed cases and 10 deaths had been reported since the start of the pandemic [3]. Between June 2020 and 01 Feb 2021, 166 health worker (HW) infections were reported in the district, including doctors, nurses, midwives, medical assistants, lab staff, pharmacists, cleaners, guards, and volunteers.

Cox's Bazar district in Bangladesh is located in the Southern region with an estimated population of 2.29 million people [4] served by 281 Health Facilities (HFs). In addition to the host population, the district has the world's largest refugee camps with approximately 907,766 Rohingya refugees [5] served by 157 camp-level HFs (90 health posts, 41 Primary Healthcare centers, 23 special facilities, and 03 field hospitals) which are managed or supported by approximately 80 health partners and non-governmental organization [6]. In response to the COVID-19 pandemic, health partners established Severe Acute Respiratory Infection Isolation and Treatment Centers (SARI ITCs). By Feb 2021, 13 SARI ITCs had been established to exclusively handle COVID-19 patients within the Rohingya camps and nearby host communities [6]. The IAR was conducted to cover all IPC response activities in the Rohingya refugee camps from January 2020 to January 2021.

The fourth Emergency Committee Meeting of International Health Regulation (IHR) held in July 2020 called upon countries to review their response to the COVID-19 pandemic for improvement [7]. Following that meeting, WHO implored countries to conduct COVID-19 IAR to help identify the strengths, gaps, and ways forward to improve the response and provided concept notes [8] and detailed guidance to countries [9].

Infection Prevention and Control (IPC) has been vital in controlling the COVID-19 pandemic and is one of the pillars of the WHO COVID-19 strategic preparedness and response plan 2020 [10]. The Office of the Civil Surgeon, humanitarian partners organized under the Health Sector, and the WHO emergency sub-office in Cox's Bazar jointly conducted a COVID-19 IPC IAR. The review had four main objectives; firstly, to provide an opportunity for health partners to share experiences with implementing IPC and analyze the ongoing COVID-19 response in Cox's Bazar by identifying challenges, best practices, and recommendations for improvement. Secondly, to compile lessons learned by various stakeholders in IPC implementation during the response to improve the current response by identifying successful and sustainable best practices and preventing recurrent errors. Thirdly, document and apply the lessons learned in IPC from the response efforts to strengthen the health system. Fourthly, to provide a basis to validate and update the COVID-19 IPC response plan for Cox's Bazar and other response and strategic plans (e.g., Health Sector) accordingly. We believe that we have had a unique opportunity to respond to the COVID-19 crisis within the ongoing Rohingya refugee crisis. Our learnings will benefit our ongoing response and future responses in similar humanitarian settings. This paper, therefore, shares the lessons learned from the IAR of the COVID-19 IPC pillar preparedness and response in Rohingya refugee camps in Cox's Bazar district Bangladesh in the form of best practices, challenges, and recommendations.

## Methods

### IAR design and area

This was an IAR of IPC interventions in HFs and SARI ITCs in Cox’s Bazar district in Bangladesh during the COVID-19 response. The approach was adopted from WHO Guidance for Conducting a Country COVID-19 IAR [11]. We conducted the review from 25 Jan to 02 February 2021. The qualitative and cross-sectional IAR was conducted at the district level looking at COVID-19 IPC response activities in HFs and SARI ITCs in the Rohingya refugee camps covering the response period from January 2020 to January 2021.

### IAR methods, selection of participants, and data collection

The IAR discussions took place in two sessions with 54 participants purposively selected from different organizations and agencies involved in the frontline implementation of IPC in the HFs and SARI ITCs. Some participants were also professionals involved in the oversight and management of the IPC interventions during the pandemic response in Cox's Bazar district. The meetings were held using a hybrid format of an online platform (Microsoft Teams) and in person, and all participants were invited through email. We used the IPC trigger questions adapted from the country COVID-19 IAR: trigger question database [12] as a discussion guide (See Additional file 1: Annex Table 1 for trigger questions used). We used the trigger questions to elicit responses on best practices, challenges, and recommendations for improvement of IPC in the COVID-19 response in Cox's Bazar district. An IAR management team consisting of facilitators, note-takers, and report writers was assembled to facilitate the meetings and collect the information shared by the participants (see Additional file 1: Annex Table 2 for a list of management teams). This team was oriented on the IAR's objectives, scope, and methodology through video conferencing with WHO experts. The orientation also included; facilitation techniques, IPC IAR trigger questions, their roles in the IAR meetings, and some examples of prior IARs.

We held two meetings with IAR participants, each lasting about 2 h and 30 min, with the online meeting having 30 participants and the in-person meeting having 24 participants (see Additional file 1: Annex Table 3 for the participants list). During the sessions, the participants were given an overview of the response, including Cox's Bazar district COVID-19 IPC response plan; the capacities before the response and those developed for and during the COVID-19 response; the actual response timeline to establish a baseline for the participants to conduct the review. We then used the COVID-19 IAR IPC trigger questions to guide participants in an open discussion to identify and analyze IPC response interventions concerning IPC best practices, IPC challenges, and recommendations for improvement.

### Data management, analysis, and ethical consideration

All meetings were recorded, and notes were taken for each session. All notes were collected from the note-takers, and recordings were transcribed verbatim. The data from the note-takers and the transcripts from the recordings were then collated and manually analyzed using content analysis under the three main themes of implementation of IPC best practices, challenges, and recommendations for improvement (see Additional file 1: Annex Table 10 for themes and subthemes).

The results were mainly summarized using text; however, quotes from the meeting transcripts that elaborately illustrated meanings or emphasized vital messages were also included.

The objectives and benefits of the IAR were clearly explained to the participants, and verbal informed consent was obtained from each participant before the meetings. All participants consented to take part in the review and to be recorded. We treated all data obtained as confidential and anonymous identifiers were used during the analysis. We restricted raw data access to only the IAR management team. Ethical approval for this IAR was foregone since the health sector coordination and government commissioned it as part of emergency response for COVID-19 outbreak response activities and quick information on subsequent actions.

## Results

The two meetings conducted for the IAR discussions collected information from the 54 participants identifying several best practices, challenges, and recommendations for improving the COVID-19 IPC response. The results are described according to three broad themes; (1) Implementation of IPC Best Practices, (2) Challenges (3) Recommendations.

### Implementation of IPC best practices

#### IPC assessments for general HFs and SARI ITCs

Predesigned, easy-to-adapt WHO IPC assessment tools and technical expertise facilitated the Health Sector and WHO IPC assessments in HFs. These assessments were conducted to determine the extent of IPC operational readiness in HFs in preparation for the COVID-19 response. Additionally, IPC operational readiness assessments were conducted before any SARI ITC opened to identify gaps before patients were received.

"The assessments were kind of needs assessments to prepare us for proper planning for interventions we needed for the response. It helped us to design activities for IPC for COVID-19 in the camps informed by evidence on the ground; otherwise, we could have just blindly implemented activities which would not yield good results." Participant 33.

#### COVID-19 IPC response plan

The Health Sector, with support from WHO, developed a response plan for COVID-19 for Cox's Bazar. The broader Health Sector COVID-19 response plan informed the IPC response plan which was widely shared with implementing partners and continues to guide the IPC interventions as emphasized by the subsequent voice.

"For us as the IPC technical working group, having a well-laid out COVID-19 IPC response plan was a key to success because it guided our response systematically, and we followed our plan well. Monthly we reviewed our plan to make sure we were on track and to see if any adjustments needed to be made for better response." Participant 10.

#### IPC Technical Working Group (TWG), leadership and coordination

The Strategic Advisory Group (SAG) of the Health Sector approved an Ad hoc IPC TWG for COVID-19 response on 20 May 2020. The group provided leadership and coordination in IPC, promoted sharing of IPC knowledge across partners, facilitated practical drills and trainings amongst HFs, and drafted IPC materials for training, monitoring, and implementing IPC interventions during the response. The IPC TWG continues to advocate for institutionalizing IPC in HFs throughout the district beyond COVID-19 pandemic.

"The IPC Technical Working Group has been a strong forum for mobilizing all health partners to improve IPC in all facilities in the camp and has led the IPC interventions. The monthly IPC technical working group meetings have always given us a chance to share experiences and the next course of action during this response." Participant 30.

The IPC TWG, through the Health Sector, provides leadership and a coordinated approach to resource mobilization and implementation of IPC interventions that helped all partners systematically and similarly support the response efforts.

#### Existence of adapted guidance documents

As a preparedness strategy, the WHO Cox's Bazar Emergency Sub Office disseminated the WHO guidance documents on IPC for COVID-19 to all Health Sector partners. The WHO guidance documents were adapted and contextualized to Cox's Bazar and Rohingya camps HFs' setting and provided to health sector partners and humanitarian workers through the health sector Google Drive document storage platform, which was accessible to all. The documents included but were not limited to guidance on; hand hygiene, respiratory hygiene, rational use of personal protective equipment (PPE), decontamination, travel, and physical gatherings.

"We got a lot of guidance documents on COVID-19 IPC from WHO and we followed them very strictly in our SARI IT and because of this, COVID-19 infection among our health workers is almost zero, and COVID-19 patients on the ward did not get infected with other diseases due to strict IPC followed by staff." Participant 3.

"Initially, we had a shortage of PPE due to overuse, so we planned to minimize unnecessary use of PPE through rational use of PPE, for example, according to the severity of patient and proper risk assessment, which reduced PPE shortage in our SARI ITC." Participant 1.

#### COVID-19 IPC trainings

WHO and the Health Sector conducted a five-days master trainers' course, which created a pool of 43 trainers (see Additional file 1: Annex table 4 for training modules covered). The master trainers then trained all HWs in the SARI ITCs and all HFs in the Rohingya refugee camps, the entire district, and humanitarian workers over three months. These trainings included lectures, practical sessions, simulations, and practical drills Trainings were conducted before SARI ITCs started admitting COVID_19 patients. The following quotes illustrate the role played by the trainings.

"The IPC master training really helped us a lot… My colleagues and I have each trained at least 500 healthcare workers in different aspects of IPC in our health facilities throughout the camp." Participant 1.

"The five days IPC master trainers' course was a very practical and effective strategy to develop human resources to fight the new disease…. we were confused and scared, we didn't know what to do, but after the training everything became clear…. we were able to set up and run big SARI ITCs like the 150 bedded SARI ITC we have here." Participant 6.

"The dry runs gave us the confidence to handle patients with less fear; as you know, it was the first time for us to manage such patients, so the dry runs helped us not to make unnecessary mistakes once we received the real patients and to reduce our chances of exposure to infections from patients." Participant 38.

#### Screening, early identification, and isolation of suspected COVID-19 patients

The HFs in Cox's Bazar introduced screening for COVID-19 signs and symptoms for all persons entering the HFs, including HWs. The availability of human resources, screening tools, and materials supported this process. Screening at HF entrances helped rapidly identify and separate suspected COVID-19 patients from other patients to reduce transmission. Early identification of suspected cases for immediate isolation and referral gave patients confidence to continue utilizing non-COVID-related essential health services.

"We have separate entrances for staff and patients and screen everyone coming to the facility. If we find that a health worker has signs and symptoms of COVID-19, we put them in isolation, and a sample is taken. We don't allow such health workers to go in to work as they could infect other health workers and patients too." Participant 1.

#### Hand hygiene at strategic points in the HFs

Almost all HFs in Cox's Bazar installed hand hygiene points at gates, waiting areas, consultation rooms, and in-patient care areas. This increased availability of supplies increased hand hygiene practice among staff, patients, and visitors as echoed here:

"We have placed hand washing points at the gate, waiting areas, and all points of care, which has increased hand washing among the patients and health workers, and hand washing, as you know, helps control the spread of many infections, not just COVID-19." Participant 16.

IAR participants reported that by increasing hand hygiene points, community trust that HFs were safe places to seek essential health services was increased. This intervention was facilitated by the availability of adequate hand hygiene supplies and the Information Education and Communication (IEC) materials provided by different partners.

"When beneficiaries saw that we were strict about hand washing in our facility, the fear reduced; they now trust our system to protect them from COVID-19, so they freely come to the facility for all services." Participant 7.

#### IPC monitoring, audit, and feedback in SARI ITCs

WHO supported partners in designing a contextualized, user-friendly daily IPC checklist and a monthly scorecard for monitoring and facilitating feedback on IPC activities in SARI ITCs. The daily checklist consisted of fourteen areas of observation vital for IPC in SARI ITCs. The IPC team in the SARI ITC conducted daily assessments using the checklist and gave immediate feedback to concerned HWs (see Additional file 1: Annex table 5 for checklist). The IPC team calculated the average scores of the daily checklist for all areas of observation at the end of the month to obtain a score for the monthly scorecard. The monthly scores were represented on HF notice boards using colors; green = good performance (80% and above), yellow = fair performance (50–79%), and red = poor performance (0–49%) (See Additional file 1: Annex table 6 for scorecard). The daily checklist helped improved IPC practice through the daily monitoring and corrections facilitated by the IPC team as they gave feedback to HWs. Simultaneously, the scorecard triggered a continuous quality improvement cycle with facilities striving to improve their monthly IPC scores. (see Additional file 1: Annex Table 7 for an example of observed changes in scores for one SARI ITC).

"Having the daily IPC checklist helped us a lot in the health facilities; whenever we found a breach, we gave immediate feedback to the health workers to correct it, and that helped us a lot in maintaining high standards of IPC in our facilities." Participant 27.

"The monthly scorecard encouraged us to work harder to improve IPC practices; whenever you see an indicator that has not turned green, for example, it is red or yellow, you work harder at it in the coming month to turn green because green is more desirable, and all staff are more motivated when they see green on the scorecard" Participant 11.

#### General masking for all patients

All patients who visited any HF to seek care were given a mask before entering the HF to reduce the risk of transmission of COVID-19. Patients with respiratory symptoms were given medical masks, while those without symptoms were provided fabric masks for source control. Adherence to mask wearing was encouraged and facilitated by an adequate supply of masks from different agencies, available IEC material on how to use masks, and HWs' commitment to teaching patients how to use the masks as expressed below.

"At our health facility, we provide free masks to all patients coming to seek care, and it helps not to spread infections of COVID-19 and other respiratory infections within our facility." Participant 26.

Additionally, the Food Security Sector in Cox's Bazar coordinated the community-wide distribution of fabric masks. Communication campaigns encouraged people to use masks in public places, including HFs.

#### Health education of patients

Patients admitted to SARI ITCs received briefings on IPC (including respiratory hygiene, hand hygiene, physical distancing, personal hygiene, and waste management) on admission and in the ward during daily IPC rounds, which was reinforced through the distribution of IEC materials. The availability of sufficient IPC staff in HFs and the IEC materials facilitated the sensitization sessions in the SARI ITCs.

"We have put up IEC materials in all visible areas in our SARI ITC, and this has helped the staff and patients to be reminded of what to do to control the spread of COVID-19 in the facility and community." Participant 34.

"Every morning, our IPC team goes to the ward to conduct IPC sensitization for patients. It has helped eliminate open spitting, littering, and poor hygiene in the wards; we also emphasize hygienic use of washrooms and proper waste segregation." Participant 25.

#### IPC supportive supervision to SARI ITCs and HFs

The WHO and IPC TWG conducted quarterly and bi-annual COVID-19 IPC supportive supervision for all SARI ITCs and all HFs, respectively, using contextualized detailed checklists (see Additional file 1: Annex Tables 8 and 9 for supportive supervision checklists). The visits were for quality control but also led to continuous improvement of IPC practices such as, but not limited to, environmental cleaning, hand hygiene, and waste management, in all HFs.

"Every time we receive colleagues who come for supportive supervision, they guide us on things that are not doing well in the facility, and we immediately improve on them; this helped us a lot." Participant 39.

#### Tracking of PPE and IPC supplies utilization in SARI ITCs

The SARI ITCs used different methods to track the consumption of IPC supplies and PPE within their facilities’, for example, Cloud-based spreadsheets and other software. All of the SARI ITCs had supportive IT systems with various tracking, forecast, and estimation methods for PPE and supplies. Tracking consumption ensured a regular supply of PPE and other supplies and timely ordering for replenishing.

"We have an Excel Google sheet to track PPE, and IPC supplies daily utilization for our health facilities. This helps us to monitor stock and procure on time to avoid stock out of PPE and IPC supplies which are essential in controlling the spread of infection." Participant 40.

#### Engineering controls in SARI ITCs and HFs

The SARI ITCs had separate entrances and exits for patients and staff, clear marking of high-risk zones (patient care areas) and low risk zones (areas without patients). All SARI ITCs had adequate human resources, signposts marking the direction of movement, and physical barriers (e.g., doors that open to only one direction) between the zones. This reduced the risk of cross-contamination between zones and amongst patients and ultimately, along with all IPC measures implemented, contributed to reduced health care associated infections (HAI) of COVID-19 among health workers, as echoed below.

"We have worked with our security staff to maintain strict use of single entrance and exists and movement of patients in the right direction and making sure patients don't cross from SARI ITC to the field hospital to avoid transferring infections between the two facilities." Participant 23.

All HFs practiced the 1-m distance between persons in waiting and triage areas, consultation rooms, wards, and all other HF spaces using several innovations including marking seats with paint, using physical barriers made from bamboo, or volunteers to instruct people on sitting and queuing arrangements.

"We maintain physical distancing in all our facilities right from waiting, screening areas, wards, and other areas, which I believe has contributed to reducing the spread of COVID-19 in our facilities and community," Participant 26.

The IPC teams worked closely with engineers to ensure that the construction of SARI ITCs had appropriate designs with IPC considerations, including; adequate space, workflow directions, separation of different zones, adequate ventilation, lighting, and waste management. It made general operations easy and safe when patient care started, as we had considered all IPC protocols.

"Our management allowed us as master trainers to work with the engineers during the construction of our SARI ITC, and they were flexible to follow our advice on the necessary spaces needed in infectious diseases hospital for proper management of patients." participant 20.

#### Health Care Waste management

Participants attested that most HFs practiced waste minimization, segregation, and disposal as the pandemic continued. This helped reduce the quantity of waste and enhanced waste handler safety. The SARI ITCs also developed innovative ways of reducing waste so that the burden of waste management was low, as echoed by HWs below.

"All fresh food and fruits are cleaned or peeled, preprocessed from the market, and only ready-to-cook food and ready-to-eat fruits are brought into the SARI ITC, which reduced the load of waste ending up in our waste management zone." Participant 24.

"We also use reusable PPE where applicable to reduce the burden of waste generated by unnecessarily using disposable PPE for which an alternative reusable PPE is available and is equally safe." Participant 19.

### Challenges

#### Frequent breakdown of incinerators

SARI ITCs reported incinerator breakdowns due to high quantities of waste generated compared to the capacity of the incinerators, improper waste segregation, and low-quality construction materials in the early phases of the COVID-19 pandemic, as echoed below.

"Our incinerators had been designed for a low capacity of waste, but with too much use of PPE came a lot of waste which was beyond the capacity of the incinerators, so they broke down often. Also, the incinerators had been built without heat-resistant materials, so they cracked and broke down quickly." Participant 50.

#### Limited PPE supply and irrational use

The global demand for PPE resulted in PPE scarcity in the market and difficulty procuring PPE from international sources. As a result, during the initial stage of the pandemic response, limited supplies led to reuse of single-use PPE and reduction of healthcare activities in the HFs as emphasized by the participant below.

"In the beginning, we had a problem of the limited stock of PPE, so it led to reusing one time use PPE like the face shield, which actually hampered our activities in the beginning." Participant 5.

In the early phase of the pandemic, PPE was overused, and was mainly driven by fear of infection and low knowledge of how to conduct a risk assessment for proper use of PPE. This exacerbated the shortage of PPE and caused unnecessary panic among health workers, patients, visitors, and communities.

"We faced challenges initially when health workers wanted us to give them three or four masks to put on during donning. Others wanted respirators to attend to mild patients where no aerosol-generating procedures were done while others wanted to put on both coverall and gown at the same time, which led to a lot of PPE going to waste" Participant 25.

#### Inconsistent adherence to IPC practices by HWs

Some HWs did not consistently follow IPC practices as provided during trainings, especially in the absence of refresher training and monitoring of actual practices. The participants highlighted that this could have resulted in HAI of COVID-19 among some HWs, especially in HFs (health posts, primary healthcare centers, and field hospitals), where there was no dedicated IPC supervisor to monitor staff adherence to IPC practices.

"When health workers are in health facilities, they follow the IPC guidance very strictly, for example, wearing masks, hand washing, physical distance, but when they leave the health facility and go to public places like malls, markets, public transport, they don't even put on a mask. It made it hard to actually trace where health workers got the infections." Participant 10.

"In the beginning, it was hard to get health workers to use the masks properly. Instead of covering the nose and mouth, the mask would be below the chin, yet we had trained on the right way to put on the masks." Participant 6.

#### Limited investigation of HW infections and HAI of COVID-19

We could not investigate all COVID-19 HW infections in the district due to inadequate human resources to administer the WHO IPC investigation tools for HW COVID-19 infections. Additionally, healthcare-associated COVID-19 infection surveillance in HFs was not conducted; yet tools like daily screening registers were availed.

"WHO has tools for investigating health worker infections; however, these have not been used by different facilities…also we have screening registers to use in the in-patient wards to follow up on signs and symptoms of COVID-19 so that if we get any suspect, a sample is taken for testing however no HF has used these registers for in-patients." Participant 10.

#### Lack of uniforms in many HFs

In many HCFs, HWs use clothes from home at work, which could potentially carry infections from the HF to their communities. This was mainly driven by unfamiliarity with the need for uniforms (e.g., medical scrubs) in HF, the limited budget for staff uniforms, and inadequate logistics and infrastructure for laundering and storage.

"We don't have working clothes for our staff, so they use their clothes for work and return to their homes with the same clothes. We know that could lead to the spread of infections from our SARI ITC to the community, but we don't have enough money to buy scrubs for our staff." Participant 13.

#### Lack of culture- and gender-adapted work uniforms and PPE in SARI ITCs

Almost all the SARI ITCs in Cox's Bazar had uniforms (e.g., medical scrubs)for staff; however, in some cases, the uniforms were not adapted for gender and culture. For example, lack of provision for head covering for female Muslim staff and low-neck lines of available uniforms leading to exposure of the upper chest created challenges for female staff. Some PPE, like surgical masks only had provisions for ear bands, yet some female health workers wear head and neck coverings, making the donning and doffing of masks difficult and leading to poor adherence to PPE.

"The scrubs did not have head and neck covering like hijab yet we needed to cover ourselves, and the design of the shirts was not comfortable for us, the ladies; it was too open, so we could not use those scrubs." Participant 20.

#### Low implementation of public health and social measures in the community

IPC interventions for reducing transmission of COVID-19 in the community, known as public health and social measures, such as hand washing, respiratory hygiene, and physical distancing, use of masks were not as widely practiced in the community, as in HFs. The identified drivers of the lack of IPC implementation in the communities were: ineffective communication of IPC interventions to the community and people’s cultural beliefs and ways of life. The comparatively low numbers of COVID-19 cases did not create a sufficient sense of urgency amongst the communities to drive behavioral changes, or compliance with the recommended IPC practices.

### Recommendations

#### Establishment of an institutionalized IPC program for Cox's Bazar district

The participants recommended that IPC be integrated throughout the health system by advocating for IPC programmes to be included within budgets, adequate staffing with trained IPC focal points, and leadership structures from the district to the lowest level of care within the health system.

#### Establishment of IPC monitoring, audit, and feedback mechanisms in all HFs

The introduction of daily IPC checklists and the monthly score card in the HFs and training of IPC focal points on implementation of these tools was identified as a good initiative for sustained and continuous improvement of IPC performance in the HFs beyond the COVID-19 pandemic.

*"We should roll out the IPC scorecard to other facilities in the camp as well; it has worked so well for the SARI ITCs… it is such a good innovation."* Participant 48.

#### IPC education and training

Training all HWs on aspects of IPC on the job, through structured training (including refresher trainings), and in the medical colleges is necessary. This requires stakeholders and health sector partners to develop a contextualized curriculum for IPC for HWs including short courses to be taught in HFs through in-services for HWs and as part of the health care education curriculum.

"WHO should work with Cox's Bazar Medical College to develop a module for teaching IPC to medical students and other health workers to build IPC capacity at a wider scale and more sustainably." Participant 47.

#### Strengthening public health and social measures in the communities

The review recommended the innovation of more robust community engagement approaches to promote IPC beyond the HF settings. There is a need for the IPC team to work closely with risk communication and community engagement experts, sociologists, psychologists, and other stakeholders to design approaches that can trigger community behavior change towards practicing recommended public health and social measures for the control of COVID-19. Recognition of message fatigue is critical in designing alternative, more creative methods to deliver common IPC messages. Close engagement with community representatives in the design of community engagement approaches is crucial, with due consideration of age, gender, cultural and diversity factors.

"We need to come up with a better strategy to change the behavior of the community…. The IPC team should work with other working groups like communicating with communities, risk communication, and community engagement to craft new strategies." Participant 54.

## Discussion

### Best practices

IPC assessments helped inform the Strategic Advisory Working group of the gaps in HFs and determine the interventions to implement for the success of the response. The needs assessments provided the evidence for targeted responses, while continuous assessments helped adjust plans to improve the response further, which is also the aim of the IAR. Similarly, the lessons learned from Ethiopia, where a risk assessment of the country for COVID-19 infections and transmission was done, verified that having done the risk assessment helped the country design appropriate interventions to reduce the importation and transmission of COVID-19 [13].

While governments needed to have COVID-19 response plans as a call for action from WHO [10], it was equally important to have a detailed step-by-step living plan for COVID-19 specific response activities, including IPC. The IPC TWG played a major role in steering the IPC-related activities and implementing the IPC response plan, which was the guiding document. Additionally, in contexts where there are many actors, as is the case with refugee settings, a coordinating platform such as the IPC TWG in Cox's Bazar helped to organize better and structure the IPC responses and maintain uniformity and standards.

Implementation of IPC is not possible without contextualized guidance documents for feasibility. In Cox's Bazar, the guidance documents were adapted from global WHO materials by a dedicated team of IPC focal persons who worked in the refugee camps and understood the context well. WHO encourages governments and HFs to adapt IPC guidance to different contexts [14] however, sometimes the capacities are limited and external experts may need to be recruited to facilitate the adaptation.

The main driver of training HWs in Cox's Bazar on IPC was to ensure compliance which was also observed in other countries in Africa and Asia [15–17]. Training of the 43 master trainers in Cox's Bazar helped in swiftly building capacity and putting in place a workforce where IPC human resource was limited, yet the task at hand was huge. The trainers reached all HWs of all cadres in the district within minimal time by cascading the training using materials centrally availed by the WHO Cox's Bazar Emergency Sub-Office. This approach proved fast, effective, and efficient for the Cox's Bazar emergency response reaching at least 3600 trained people within three months [18]. Such trainings should consider utilizing trainers with a certain level of authority or leadership (formal or informal) among their peers who can cascade the training to their respective jurisdictions. Training trainers with limited authority and organizational support to cascade the training will not achieve the desired result of passing on knowledge to others.

Hand hygiene, use of masks, physical distancing, and screening are some of the IPC measures recommended by WHO for control of COVID-19 transmission in HFs [9]. These were comprehensively implemented in Cox's Bazar, and we believe they contributed to the reduction of infections in general, not only for COVID-19, because similar interventions have reported a decrease of HAIs elsewhere [19–21]. Implementing the interventions was possible due to adequate funding from different partners and donors; however, this may be difficult to implement in settings without similar resources. Although the main aim of increased hand hygiene, provision of masks, and screening before entering HFs is to control COVID-19 transmission, these interventions resulted in unintended positive effects, including increased trust from the community and sustained essential healthcare seeking behaviours by refugees. While utilization of essential health services was reduced briefly in the early phases of the pandemic in the Rohingya camps [22], it was not as much and not for a prolonged period as observed globally [23–26]. The IPC interventions built the confidence and trust amongst the community that HFs were trying their best to control the spread of COVID-19 hence reducing fear among patients of HAIs as they sought care.

IPC monitoring, audit, and feedback is one element of the core components of IPC programmes [14]. Auditing helps with the timely identification and correction of any IPC gaps in HFs and with HW practices for improvement, as observed in some hospital contexts [27]. Consistent monitoring, audits, and feedback using different tools and methods, including the daily IPC checklists and monthly scorecards such as the ones used in Cox's Bazar context, requires dedicated IPC staff, training, and supportive leadership.

### Challenges

The global demand for PPE resulted in PPE scarcity in the market and difficulty procuring PPE from international sources due to travel restrictions. In addition, there was also irrational use of PPE by HW in the early phase of the pandemic. It is important to continuously build the capacity of HWs IPC knowledge, beginning with training in academia and continuing with on-the-job and refresher trainings, thus enabling them to follow IPC practices including appropriate use of PPE according to the context and situation. Most importantly, risk communication and community engagement experts play an integral role in helping people in the community understand the disease transmission to avoid unnecessary use of PPE by HWs and reduce fear, as also recommended by other studies [26].

Risk assessment and management of exposure of HWs in the context of COVID-19 using WHO tools help the IPC focal points and HF management to determine IPC gaps that may increase HW exposures to COVID-19 within the HF and work towards finding solutions [28]. In Cox's Bazar, however, due to lack of human resource training on the tool and the absence of a system for, the investigation of the risk of exposure of HWs in the HFs and SARI ITCs, this was not done comprehensively.

Although budgets are limited, appropriate work uniforms and PPE should be prioritized for HWs in all healthcare settings which are ideally adapted to culture and gender. Work uniforms and PPE that are not culture and gender sensitive will not be appropriately used leading to potential breaches of IPC. For example, female Muslim staff whose uniforms lacked neck and head covering used their head coverings while attending to patients and then took the same cloth home, potentially transferring infections to the home. It is therefore important for health systems to study the culture, adapt working uniforms, and procure PPE to suit particular contexts.

According to IAR participants, the limited implementation of IPC practices, for example, the use of masks and physical distancing in the community, could have been driven by culture and beliefs, which aligns with studies across the globe [29–31]. For example, the women have a culture of wearing a face veil which they believed was sufficient covering, reducing their perceived need to wear a facemask to prevent COVID-19 transmission. Also, the comparatively limited number of detected COVID-19 cases in the different communities did not create sufficient perceived susceptibility to drive people towards new behavior, as one of the variables in the health belief model suggests [32].

## Conclusions

The Intra Action Review for IPC in the COVID-19 response was instrumental in identifying gaps in the operational response to trigger rapid adjustments in the Cox's Bazar refugee camps and lessons that could be used elsewhere in similar settings.

IPC monitoring tools and continuous training adapted to the local context provide critical support in promoting consistent IPC implementation.

Proper coordination and leadership at all levels are essential in implementing IPC measures for the ongoing response to COVID-19. IPC for COVID-19 and other infectious diseases in emergency situations and refugee settings with many actors involved in health-care delivery can only be successful with highly coordinated leadership, efficient resource mobilization, and close supervision throughout the entire response.

Purposeful and consistent engagement with HWs and the community, including appreciating cultural practice, is critical to the success of implementation and adaption of IPC measures.

## Supplementary Information

**Additional file 1.**

## Data Availability

The datasets used and analyzed during the current study are available from the corresponding author upon reasonable request.

## Abbreviations

BDRCS: Bangladesh Red Crescent Society
CME: Continuous Medical Education
FH: Food for Hungry
HAIs: Healthcare-associated Infections
HFs: Health Facilities
HWs: Health Workers
HCWM: Healthcare Waste Management
IAR: Intra-Action Review
ICDDR, B: International Centre for Diarrheal Diseases Research, Bangladesh
IEC: Information Education and Communication
IFRC: International Federation of the Red Cross and the Red Crescent
IOM: International Organization of Migration
IPC: Infection Prevention and Control
ITC: Isolation and Treatment Centre
MHPSS WG: Mental Health and psychosocial support Working Group
MoHFW: Ministry of Health and Family Welfare
MoHFWCC: Ministry of Health and Family Welfare Coordination Cell
MSF: Médecins Sans Frontières
MTI: Medical Teams International
PPE: Personal Protective Equipment
RI: Relief International
RRRC: Refugee Relief and Repatriation Commissioner
SARI: Severe Acute Respiratory Infection
SCI: Save the Children International
TWG: Technical Working Group
UNFPA: United Nations Population Fund
UNHCR: United Nations High Commissioner for Refugees
UNICEF: United Nations International Children's Emergency Fund
WHO: World Health Organization
