# Supplementary material for: Infection prevention and control for COVID-19 response in the Rohingya refugee camps in Bangladesh: an intra-action review
Source: Int J Equity Health. 2023 Jun 6;22:111. doi: 10.1186/s12939-023-01926-2 (PMC10241551; doi:10.1186/s12939-023-01926-2)
Supplement: Supplementary file 1 — Additional file 1. [file 12939_2023_1926_MOESM1_ESM.docx]

Annex 1: Additional file for Infection Prevention and Control for COVID-19 response in the Rohingya refugee camps: an Intra-Action Review

Table 1: Trigger questions used for IPC IAR

| **SN** | **Question** |
| --- | --- |
| 1 | What were the existing plans, SOPs and preparedness measures for infection prevention and control at healthcare facilities, conveyances, PoEs, schools, workplaces, entertainment setting and other places where people gather, before the first case of COVID-19 was detected in the district? |
| 2 | How was the functioning of the IPC programme at district and health facility levels (e.g., according to the WHO IPC core components guidelines)? How frequently were compliance and effectiveness evaluated and published? |
| 3 | What was the availability of national and subnational stockpiles of PPE for healthcare staff and other frontline workers before the first case of COVID-19 was detected in the district? |
| 4 | What were the existing plans and SOPs for waste management within healthcare and laboratory settings? |
| 5 | What were the human resources responsible for IPC and trained in IPC in the district? What was the frequency of IPC training in the district? |
| 6 | How were IPC (e.g., availability of triage and isolation facilities, availability of PPEs) enforced and monitored in healthcare facilities in the district? |
| 7 | What was the process for managing and distributing sufficient and appropriate resources including hand hygiene supplies and PPEs, so they are readily available for frontline health workers (e.g., doctors, nurses, laboratory personnel) and other essential workers to ensure a safe environment for the management of suspected or confirmed COVID-19 cases? |
| 8 | How was COVID-19 waste and contaminated items managed in healthcare (e.g., hospitals, clinics, laboratories) and community settings? What were the resources available for managing these during the COVID-19 outbreak? |
| 9 | What types of new legislations, policies or advisories were put in place for mandatory IPC measures (e.g., mandatory use of masks, physical distancing) in community settings during the COVID-19 outbreak? |
| 10 | How was the coordination with other sectors (including the private sector) to ensure the implementation of IPC measures in healthcare and community settings? How about for WASH activities? |
| 11 | Are all policies, plans and measures put in place during the COVID-19 response taking gender, equity and human rights into consideration? |
| 12 | What were the main challenges experienced regarding implementing IPC measures, waste, and water and sanitation management at healthcare and laboratories settings during the COVID-19 outbreak? How about community settings? Why was it challenging? |
| 13 | What actions were taken that allowed for IPC measures, waste, and water and sanitation management to be effectively implemented at healthcare and laboratory settings during the COVID-19 outbreak? How about community settings? |
| 14 | What new technologies or innovations were implemented to efficiently implement IPC measures at healthcare and laboratory settings during the COVID-19 outbreak? How about community settings? |
| 15 | What limitations were identified when implementing new technologies or innovations for implementing IPC measures during the COVID-19 outbreak? |
| 16 | What were the challenges for water and sanitation in healthcare and community settings during the COVID-19 outbreak? |
| 17 | What best practices can be identified for ensuring water and sanitation in rural healthcare and community settings during the COVID-19 outbreak? |
| 18 | What were the overall strengths and weaknesses of IPC, waste, and water and sanitation management measures put in place during the COVID-19 outbreak? |
| 19 | What best practices can be identified for preventing infection among health workers, visitors and other patients in healthcare settings? How about in community settings? |
| 20 | Which challenges remained with respect to gender, equity and human rights? |

**Table 2: IAR management team**

| **SN** | **Name** | **Role in IAR management** | **Designation** |
| --- | --- | --- | --- |
| 1 | Rebecca Racheal Apolot | Facilitator and Report writer | IPC Consultant |
| 2 | Debashish Paul | Facilitator | Laboratory Officer |
| 3 | Dr. S M Niaz Mowla | Facilitator | Communicable Diseases Officer |
| 4 | Dr. Ssentamu Simon Kaddu | Facilitator and report writer | Emergency Public Health Consultant |
| 5 | Dr. Egmond Samir Evers | Facilitator | Health Sector Coordinator |
| 6 | Dr. Aritra Das | Note taker | IPC focal point FH/UNHCR SARI ITC |
| 7 | Dr. Abeed Hasan | Note taker | Clinical Supervisor, IOM EPR |
| 8 | Dr. Alam Muhammad Irfanul | Note taker | Healthcare Waste Management Consultant |
| 9 | Tatiana Almeida | Note taker | External Communications Officer |
| 10 | S M Asif Sazed | Note taker | Information management officer |

Table 3: List of IAR participants

| SN | Name | Organization | Designation |
| --- | --- | --- | --- |
| 1 | Dr. Kabir Tanjila | Save the children International (SCI) | IPC Supervisor |
| 2 | Dr. MOWLA S M Niaz | World Health organization (WHO) | Communicable Diseases Officer |
| 3 | Mesbha Uddin Ahmed | Bangladesh Red Crescent Society | Medical Officer |
| 4 | Neoaz Tareq | Save the children International (SCI) | WASH manager |
| 5 | Paul Debashish | World Health organisation (WHO) | Laboratory Officer |
| 6 | Dr. Rayamajhi Rajan | World Health organisation (WHO) | Public Health Officer |
| 7 | Dr. Robert Lukwata | Food for Hungry/Medical Teams International (FH/MTI) | Program Director (Health) |
| 8 | Secondo John Loku | World Health organization (WHO) | WASH Officer |
| 9 | Dr. Ssentamu Simon Kaddu | World Health organization (WHO) | Emergency Public Health Consultant |
| 10 | Dr. Taznuva Sultana Tonni | United Nations High Commissioner for Refugees (UNHCR) | Public Health Associate |
| 11 | Dr. Abeed Hasan | International Organisation of Migration (IOM) | Clinical Supervisor, IOM EPR |
| 12 | Dr. Abu Sayum | International Organisation of Migration (IOM) | Clinical Supervisor, Camp 20Ext SARI ITC |
| 13 | Dr. Mohammad Arif Hossain | Cox’s Bazar Sadar Hospital | IPC Focal point/ Assistant prof. of Surgery |
| 14 | Dr. Sabbir Ahmed | World Health organization (WHO) | District coordinator-TB |
| 15 | Dr. ALAM, Muhammad Irfanul | World Health organization (WHO) | HCWM Consultant |
| 16 | Dr. Aritra Das | Food for Hungry/Medical Teams International (FH/MTI) | IPC focal point FH/UNHCR SARI ITC |
| 17 | Dr. Marina Binte Alam | International Organisation of Migration (IOM) | Medical Officer, Ukhiya HC |
| 18 | Dr. Hamim Tassdik | International Organisation of Migration (IOM) | Clinical Supervisor, Camp 20Ext SARI ITC |
| 19 | Rebecca Racheal Apolot | World Health organization | IPC Consultant |
| 20 | Mohammad Asad | Relief International (RI) | Laboratory Coordinator |
| 21 | Dr. Sukanya Preeti Usha | Bangladesh Red Crescent Society (BDRCS) | Medical Coordinator-BDRCS SARI ITC |
| 22 | Motiur Rahman | UNICEF/ICDDRB | Manager, IPC-UNICEF/ICDDRB SARI ITC |
| 23 | Dr. Md. Abdul Kader | BDRCS | IPC focal (Medical officer)-BDRCS SARI ITC |
| 24 | Saheen Hossain | UNICEF/ICDDRB | Officer, IPC IPC-UNICEF/ICDDRB SARI ITC |
| 25 | Md. Masud Sohel | Relief International | IPC supervisor RI/UNHCR SARI ITC |
| 26 | AKM Anisuzzaman | Care Bangladesh | Project manager |
| 27 | Dr. Sumaya Tasnim | International Organisation of Migration (IOM) | IPC supervisor Camp 24 SARI ITC |
| 28 | Tatiana Almeida | World Health Organisation (WHO) | External Communications Officer |
| 29 | Alam Jahangir | International Organisation of Migration (IOM) | Project Assistant |
| 30 | Dr. Jonayed Sidiki | Prantic/Obat helpers | Clinic manager |
| 31 | Dr. Kai von Harbou | World Health organisation (WHO) | Head of Sub Office |
| 32 | Dr. Egmond Evers | WHO/Health sector | Health sector coordinator |
| 33 | Dr. Moonmoon Munisha Dey | Food for Hungry/Medical Teams International (FH/MTI) | IPC focal point FH/UNHCR SARI ITC |
| 34 | Dr. Feroz Khan | World Health organisation (WHO) | National Profession Officer-Epidemiology |
| 35 | Dr. Tanjida Basanti | Save the Children International (SCI) | SRH manager |
| 36 | Jessica Holterhof | WHO/health sector | Partner Coordination Consultant |
| 37 | S M Asif Sazed | WHO/health sector | Information management officer |
| 38 | Dr. Mominul Haque Munna | International Rescue Committee (IRC) | Senior Manager - Health |
| 39 | Dr. Allen Gidraf Kahindo Maina | United Nations High Commissioner for Refugees (UNHCR) | Senior Public Health Officer |
| 40 | Dr. Marcella Nyawara | International Organisation of Migration (IOM) | COVID-19 Migration Health Office |
| 41 | Sayantan Chowdhury | UNFPA/SRH-WG | Maternal Health officer |
| 42 | Thok Johnson Gony | Médecins Sans Frontières (MSF) | Medical Coordinator |
| 43 | Donald Sonne Kazungu | Médecins Sans Frontières (MSF) | Medical Coordinator |
| 44 | Khairul Islam | Government of Bangladesh (MoHFW-CC) | Field coordinator-Operations |
| 45 | Dr. ASM Isthiak | Government of Bangladesh (MoHFW-CC) | Field coordinator |
| 46 | Dr. Toha Bhuiyan | Government of Bangladesh (RRRC) | Health coordinator |
| 47 | Dr. Yulia Widiati | United Nations International Children’s Emergency Fund (UNICEF) | Team lead-Health |
| 48 | Dr. Abdullah Al-Noman | Save the Children International (SCI) | Senior Program manager |
| 49 | D.r Md. Mahmudul Hasan | BRAC | Advisor health and nutrition |
| 50 | Dr. Bayezeed Mostaque/ | Bangladesh Red Crescent Society (BDRCS) | Health and PSS manager |
| 51 | Dr. Somen Palit | International Federation of the Red Cross and the Red Crescent (IFRC) | Health manager |
| 52 | Dr. Md.Mazharul Islam Zion | World Health organisation (WHO) | Immunization coordinator |
| 53 | Kenny R | International Organisation of Migration (IOM) | MHPSS WG |
| 54 | Dr. Sayem Chowdhury | Save the Children International (SCI) | Clinic Manager |

**Table 4: Modules covered in the COVID-19 IPC master trainers course**

| **Topic** | **Title** |
| --- | --- |
| Module 01 | Introduction to COVID-19: IPC expert training and adult learning |
| Module 02 | Introduction to infection prevention and control |
| Module 03 | Introduction to COVID-19 and COVID-19 infection prevention and control |
| Module 04 | Hand hygiene |
| Module 05 | Personal Protective Equipment (PPE) overview |
| Module 06 | Respiratory hygiene |
| Module 07 | Setting up the healthcare facility for COVID-19 |
| Module 08 | Screening and introduction to isolation |
| Module 09 | Cleaning disinfecting and waste Management |
| Module 10 | Rational use of PPE |
| Module 11 | Isolation and SARI ITC setup |
| Module 12 | mistakes in using Personal Protective Equipment (PPE) |
| Module 13 | Quality assurance of IPC practices |
| Module 14 | Sterilization and high-level disinfection (HLD) |
| Module 15 | IPC for conveyance team |

**Table 5: Daily IPC checklist for SARI ITCs**

|  | **Area under observation** | **Score**  **Yes=1 No=0** | **Action for improvement** | | | |
| --- | --- | --- | --- | --- | --- | --- |
| **A** | **SCREENING, WAITING AND TRIAGE AREA** |  |  | | |  |
| 1 | Screening tools functional (registers, thermometer) |  |  | | |  |
| 2 | Clean and dry (no visible dirt/litter/wetness) & well ventilated |  |  | | |  |
| 3 | PPE Stock for HCW present (gown, mask, gloves, face shield) |  |  | | |  |
| 4 | 1 m kept between patients, patients & HCW |  |  | | |  |
| 5 | Hand washing facilities available (soap, water/ ABHR) |  |  | | |  |
| 6 | Hand wash basin clean |  |  | | |  |
|  | **Total score** |  | 0-3 | 4 | 5-6 | **99** |
| **B** | **PPE DONNING AREA** |  |  | | |  |
| 1 | Clean and dry (no visible dirt/litter/wetness) & well ventilated |  |  | | |  |
| 2 | Donning supervised for all shifts (donning poster available) |  |  | | |  |
| 3 | Enough PPE stock per shift (gown, masks, glove-different sizes, respirators, face shield/goggles) |  |  | | |  |
| 4 | Hand hygiene materials available |  |  | | |  |
| 5 | Hand wash basin clean |  |  | | |  |
|  | **Total score** |  | 0-2 | 3 | 4-5 | **99** |
| **C** | **PATIENT WARDS** |  |  | | |  |
| 1 | Clean, dry and tidy (floor, walls and roof) & well ventilated |  |  | | |  |
| 2 | Hand washing facilities available (soap, water)/ABHR available at point of care |  |  | | |  |
| 3 | Lights, switches, patients’ cupboards, sinks, IV stands, and other fixtures clean |  |  | | |  |
| 4 | Beds, linen, screens, bed pans and other containers clean |  |  | | |  |
| 5 | Waste segregation bins (available, clean, liners, covers < 3/4 full) |  |  | | |  |
| 6 | Sharps container (on trolley or next to patient beds & < 3/4 full) |  |  | | |  |
| 7 | Medication preparation area and nursing trolley are clean and well organized |  |  | | |  |
| 8 | Biomedical equipment visibly clean (glucometers, pulsometers, oxygen cylinders, concentrators, etc) |  |  | | |  |
| 9 | Urinary drainage bags off floor |  |  | | |  |
| 10 | Intravascular devices – correct dressings |  |  | | |  |
| 11 | Date of insertion of IV catheters is registered |  |  | | |  |
| 12 | IV infusion sets are dated and discard (maximum) after 24 hours |  |  | | |  |
| 13 | Open ampoules are immediately discarded. Open vials are properly labelled (volume, date, time if MDV) |  |  | | |  |
|  | **Total score** |  | 0-7 | 8-10 | 11-13 | **99** |
| **D** | **Laboratory** |  |  | | |  |
| 1 | The lab is visibly clean (floor, walls, work tables, machines and all fittings), dry and ventilated |  |  | | |  |
| 2 | Hand wash station with clean water, soap and one-use paper towel and ABHR is also available |  |  | | |  |
| 3 | Waste properly segregated in the correct containers (including sharps container) and < ¾ of full |  |  | | |  |
| 4 | PPE is available (gloves, gowns, mask, respirators, face shield) |  |  | | |  |
| 5 | All the staff wear the proper working clothes (scrubs, close shoes/boots). Staff don’t wear jewelry, rings or bracelets in their hands |  |  | | |  |
|  | **Total score** |  | **0-2** | **3** | **4-5** | **99** |
| **E** | **PPE DOFFING AREA** |  |  | | |  |
| 1 | Clean, dry (no visible dirt/litter/wetness) & well ventilated |  |  | | |  |
| 2 | Doffing supervised for all shifts (doffing poster must be available) |  |  | | |  |
| 3 | PPE waste containers available & < ¾ full |  |  | | |  |
| 4 | Hand hygiene materials (water, soap, ABHR) |  |  | | |  |
|  | **Total score** |  | 0-2 | 3 | 4 | **99** |
| **F** | **STAFF RESTING AREA** |  |  | | |  |
| 1 | Clean and dry and tidy (no visible dirt/litter/wetness) &well ventilated |  |  | | |  |
| 2 | Hand hygiene materials (water, soap/ABHR) |  |  | | |  |
| 3 | Hand wash basin clean |  |  | | |  |
| 4 | Provision for staff to seat at 1m distance |  |  | | |  |
| 5 | Furniture and other fixtures clean |  |  | | |  |
| 6 | Closed waste bin |  |  | | |  |
| 7 | Disposal paper towels/tissues |  |  | | |  |
|  | **Total score** |  | 0-3 | 4-5 | 6-7 | **99** |
| **G** | **KITCHEN** |  |  | | |  |
| 1 | Clean and dry (no visible dirt/litter/wetness) & well ventilated |  |  | | |  |
| 2 | Kitchen trolley and utensils clean |  |  | | |  |
| 3 | Cooking equipment clean (no visible dirt-in and outside) |  |  | | |  |
| 4 | Fridge clean (no visible dirt-in and outside) |  |  | | |  |
| 5 | Hand washing facilities available (soap, water) |  |  | | |  |
| 6 | Hand wash and kitchen sinks are clean |  |  | | |  |
| 7 | Kitchen staff put on apron and head cover |  |  | | |  |
|  | **Total score** |  | 0-3 | 4-5 | 6-7 | **99** |
| H | **BATHROOM/TOILETS** |  |  | | |  |
| 1 | Bathrooms clean (no visible dirt/litter-walls, floors, roof, fixtures, bucket, water pot, doors, soap case) |  |  | | |  |
| 2 | Toilets clean (no visible dirty-walls, floor, roof seat, squat pan, hand wash basin, doors, bucket, water pot) |  |  | | |  |
| 3 | Hand washing facilities (soap and water, instructions) |  |  | | |  |
| 4 | Cleaning roster up to date and duly signed |  |  | | |  |
|  | **Total score** |  | **0-2** | **3** | **4** | **99** |
| **I** | **DECONTAMINATION AREA** |  |  | | |  |
| 1 | Clean and dry and tidy (no visible dirt/litter-walls, floors, roof, fixtures, doors) & ventilated |  |  | | |  |
| 2 | Enough different concentrations of chlorine prepared and labelled including mixing and discarding time recorded |  |  | | |  |
| 3 | Staff put on PPE (gown/coverall, boots, heavy duty gloves, mask, googles/face shield, heavy duty apron,) |  |  | | |  |
| 4 | Job aids (guides, SOPs, etc) for decontamination displayed and followed (linen, PPE, Equipment) |  |  | | |  |
| 5 | Uni directional flow of linen and equipment followed |  |  | | |  |
| 6 | All machines clean (washing, drying, autoclave-inside and outside) |  |  | | |  |
| 7 | Basins, buckets, mops are clean disinfected and stored dry |  |  | | |  |
| 8 | Hand washing facilities available (soap and water) |  |  | | |  |
| 9 | Washing sinks clean |  |  | | |  |
|  | **Total score** |  | **0-5** | **6-7** | **8-9** | **99** |
| **J** | **STORAGE AREA** |  |  | | |  |
| 1 | Clean and dry and tidy (no visible dirt/litter-walls, floors, roof, doors and other fixtures) &well ventilated |  |  | | |  |
| 2 | All items separately and neatly stored and labelled |  |  | | |  |
| 3 | Carts/buckets for transferring items from store clean |  |  | | |  |
| 4 | Closed waste bin |  |  | | |  |
|  | **Total score** |  | **0-2** | **3** | **4** | **99** |
| K | **PHARMACY** |  |  | | |  |
| 1 | Clean and dry and tidy (no visible dirt/litter-walls, floors, roof, fixtures, doors and other fixtures) & well ventilated |  |  | | |  |
| 2 | Closed waste bins for different wastes |  |  | | |  |
| 3 | Hand hygiene Materials (soap, water/ABHR) |  |  | | |  |
| 4 | Disposable paper towels available |  |  | | |  |
|  | **Total score** |  | **0-2** | **3** | **4** | **99** |
| **L** | **WASTE MANAGEMENT AREA** |  |  | | |  |
| 1 | Waste kept as segregated at final disposal |  |  | | |  |
| 2 | Incinerator functional |  |  | | |  |
| 3 | Hand washing facilities available (soap, water) |  |  | | |  |
| 4 | Hand wash basin clean |  |  | | |  |
| 5 | Staff put on PPE (gown/coverall, boots, heavy duty gloves, mask, googles/face shield) |  |  | | |  |
|  | **Total score** |  | **0-2** | **3** | **4-5** | **99** |
| **M** | **STAFF HEALTH** |  |  | | |  |
| 1 | Staff screened for COVID-19 symptoms |  |  | | |  |
| 2 | All staff wear clean uniform/scrubs |  |  | | |  |
| 3 | All staff wear closed shoes |  |  | | |  |
| 4 | All staff use PPE correctly |  |  | | |  |
|  | **Total score** |  | **0-2** | **3** | **4** | **99** |
| **N** | **Personal Protective Equipment (PPE)** |  |  | | |  |
| 1 | PPE supplies are stored off the floor and in a dry place |  |  | | |  |
| 2 | Daily stock taking done |  |  | | |  |
| 3 | Different PPE stored and labelled for easy identification |  |  | | |  |
| 4 | PPE stored in Clean and dry & well-ventilated room |  |  | | |  |
|  | **Total score** |  | **0-2** | **3** | **4** | **99** |

**Table 6: Monthly IPC score card for SARI ITCs in the Cox’s Bazar**

|  | **Area under observation** | ***Average score colour code: Red = poor performance, Yellow = fair performance, Green = Good performance and Gray = not applicable*** | | | | | | | | | | | |
| --- | --- | --- | --- | --- | --- | --- | --- | --- | --- | --- | --- | --- | --- |
|  |  | **Months** | | | | | | | | | | | |
|  |  |  |  |  |  |  |  |  |  |  |  |  |  |
| A | Screening, Waiting and Triage Area |  |  |  |  |  |  |  |  |  |  |  |  |
| B | PPE Donning Area |  |  |  |  |  |  |  |  |  |  |  |  |
| C | Patient Wards |  |  |  |  |  |  |  |  |  |  |  |  |
| D | Laboratory |  |  |  |  |  |  |  |  |  |  |  |  |
| E | PPE Doffing Area |  |  |  |  |  |  |  |  |  |  |  |  |
| F | Staff Resting Area |  |  |  |  |  |  |  |  |  |  |  |  |
| G | Kitchen |  |  |  |  |  |  |  |  |  |  |  |  |
| H | Bathroom/toilets |  |  |  |  |  |  |  |  |  |  |  |  |
| I | Decontamination Area |  |  |  |  |  |  |  |  |  |  |  |  |
| J | Storage Area |  |  |  |  |  |  |  |  |  |  |  |  |
| k | Pharmacy |  |  |  |  |  |  |  |  |  |  |  |  |
| L | Waste Management Area |  |  |  |  |  |  |  |  |  |  |  |  |
| M | Staff Health |  |  |  |  |  |  |  |  |  |  |  |  |
| N | Personal Protective Equipment (PPE) |  |  |  |  |  |  |  |  |  |  |  |  |

**Table 7: One year period (September 2020 to August 2021) IPC monthly score card for SARI ITC ‘X’ in the Rohingya refugee camps of Cox’s Bazar.**

| **Areas under observation** | **2020** | | | | **2021** | | | | | | | |
| --- | --- | --- | --- | --- | --- | --- | --- | --- | --- | --- | --- | --- |
|  | **Sep** | **Oct** | **Nov** | **Dec** | **Jan** | **Feb** | **Mar** | **Apr** | **May** | **Jun** | **Jul** | **Aug** |
| Screening, waiting and triage area |  |  |  |  |  |  |  |  |  |  |  |  |
| PPE donning area |  |  |  |  |  |  |  |  |  |  |  |  |
| Patient wards |  |  |  |  |  |  |  |  |  |  |  |  |
| Laboratory |  |  |  |  |  |  |  |  |  |  |  |  |
| PPE doffing area |  |  |  |  |  |  |  |  |  |  |  |  |
| Staff resting area |  |  |  |  |  |  |  |  |  |  |  |  |
| Kitchen/dining |  |  |  |  |  |  |  |  |  |  |  |  |
| Toilets and shower room |  |  |  |  |  |  |  |  |  |  |  |  |
| Decontamination area |  |  |  |  |  |  |  |  |  |  |  |  |
| Storage area |  |  |  |  |  |  |  |  |  |  |  |  |
| Pharmacy/pharmacy store |  |  |  |  |  |  |  |  |  |  |  |  |
| Waste management area |  |  |  |  |  |  |  |  |  |  |  |  |
| Staff health & safety |  |  |  |  |  |  |  |  |  |  |  |  |
| Personal protective equipment (PPE) and supplies |  |  |  |  |  |  |  |  |  |  |  |  |

**Table 8: Quarterly IPC supportive supervision checklist for SARI ITCs in Rohingya refugee camps in Cox’s Bazar.**

| Implementing Partner(s)____________Facility name and UID___________ Visiting team ________________Date of visit___________ | | | |
| --- | --- | --- | --- |
|  | | | |
|  | **Standard per area under observation** | **Findings** | **Suggested actions for improvement** |
| **A** | **SCREENING, WAITING AND TRIAGE AREA** |  |  |
| 1 | Screening tools functional (registers, thermometer) |  |  |
| 2 | Clean and dry (no visible dirt/litter/wetness) & well ventilated |  |  |
| 3 | PPE Stock for HCW present (gown, mask, gloves, face shield) |  |  |
| 4 | 1 m kept between patients, patients & HCW |  |  |
| 5 | Hand washing facilities available (soap, water/ ABHR) |  |  |
| 6 | Hand wash basin clean |  |  |
| **B** | **PPE DONNING AREA** |  |  |
| 1 | Clean and dry (no visible dirt/litter/wetness) & well ventilated |  |  |
| 2 | Donning supervised for all shifts (donning poster available) |  |  |
| 3 | Enough PPE stock per shift (gown, masks, glove-different sizes, respirators, face shield/goggles) |  |  |
| 4 | Hand hygiene materials available |  |  |
| 5 | Hand wash basin clean |  |  |
| **C** | **PATIENT WARDS** |  |  |
| 1 | Clean, dry and tidy (floor, walls and roof) & well ventilated |  |  |
| 2 | Hand washing facilities available (soap, water)/ABHR available at point of care |  |  |
| 3 | Lights, switches, patients’ cupboards, sinks, IV stands, and other fixtures clean |  |  |
| 4 | Beds, linen, screens, bed pans and other containers clean |  |  |
| 5 | Waste segregation bins (available, clean, liners, covers < 3/4 full) |  |  |
| 6 | Sharps container (on trolley or next to patient beds & < 3/4 full) |  |  |
| 7 | Medication preparation area and nursing trolley are clean and well organized |  |  |
| 8 | Biomedical equipment visibly clean (glucometers, pulsometers, oxygen cylinders, concentrators, etc) |  |  |
| 9 | Urinary drainage bags off floor |  |  |
| 10 | Intravascular devices – correct dressings |  |  |
| 11 | Date of insertion of IV catheters is registered |  |  |
| 12 | IV infusion sets are dated and discard (maximum) after 24 hours |  |  |
| 13 | Open ampoules are immediately discarded. Open vials are properly labelled (volume, date, time if MDV) |  |  |
| **D** | **Laboratory** |  |  |
| 1 | The lab is visibly clean (floor, walls, work tables, machines and all fittings), dry and ventilated |  |  |
| 2 | Hand wash station with clean water, soap and one-use paper towel and ABHR is also available |  |  |
| 3 | Waste properly segregated in the correct containers (including sharps container) and < ¾ of full |  |  |
| 4 | PPE is available (gloves, gowns, mask, respirators, face shield) |  |  |
| 5 | All the staff wear the proper working clothes (scrubs, close shoes/boots). Staff don’t wear jewelry, rings or bracelets in their hands |  |  |
| **E** | **PPE DOFFING AREA** |  |  |
| 1 | Clean, dry (no visible dirt/litter/wetness) & well ventilated |  |  |
| 2 | Doffing supervised for all shifts (doffing poster must be available) |  |  |
| 3 | PPE waste containers available & < ¾ full |  |  |
| 4 | Hand hygiene materials (water, soap, ABHR) |  |  |
| **F** | **STAFF RESTING AREA** |  |  |
| 1 | Clean and dry and tidy (no visible dirt/litter/wetness) &well ventilated |  |  |
| 2 | Hand hygiene materials (water, soap/ABHR) |  |  |
| 3 | Hand wash basin clean |  |  |
| 4 | Provision for staff to seat at 1m distance |  |  |
| 5 | Furniture and other fixtures clean |  |  |
| 6 | Closed waste bin |  |  |
| 7 | Disposal paper towels/tissues |  |  |
| **G** | **KITCHEN** |  |  |
| 1 | Clean and dry (no visible dirt/litter/wetness) & well ventilated |  |  |
| 2 | Kitchen trolley and utensils clean |  |  |
| 3 | Cooking equipment clean (no visible dirt-in and outside) |  |  |
| 4 | Fridge clean (no visible dirt-in and outside) |  |  |
| 5 | Hand washing facilities available (soap, water) |  |  |
| 6 | Hand wash and kitchen sinks are clean |  |  |
| 7 | Kitchen staff put on apron and head cover |  |  |
| H | **BATHROOM/TOILETS** |  |  |
| 1 | Bathrooms clean (no visible dirt/litter-walls, floors, roof, fixtures, bucket, water pot, doors, soap case) |  |  |
| 2 | Toilets clean (no visible dirty-walls, floor, roof seat, squat pan, hand wash basin, doors, bucket, water pot) |  |  |
| 3 | Hand washing facilities (soap and water, instructions) |  |  |
| 4 | Cleaning roster up to date and duly signed |  |  |
| **I** | **DECONTAMINATION AREA** |  |  |
| 1 | Clean and dry and tidy (no visible dirt/litter-walls, floors, roof, fixtures, doors) & ventilated |  |  |
| 2 | Enough different concentrations of chlorine prepared and labelled including mixing and discarding time recorded |  |  |
| 3 | Staff put on PPE (gown/coverall, boots, heavy duty gloves, mask, googles/face shield, heavy duty apron,) |  |  |
| 4 | Job aids (guides, SOPs, etc) for decontamination displayed and followed (linen, PPE, Equipment) |  |  |
| 5 | Uni directional flow of linen and equipment followed |  |  |
| 6 | All machines clean (washing, drying, autoclave-inside and outside) |  |  |
| 7 | Basins, buckets, mops are clean disinfected and stored dry |  |  |
| 8 | Hand washing facilities available (soap and water) |  |  |
| 9 | Washing sinks clean |  |  |
| **J** | **STORAGE AREA** |  |  |
| 1 | Clean and dry and tidy (no visible dirt/litter-walls, floors, roof, doors and other fixtures) &well ventilated |  |  |
| 2 | All items separately and neatly stored and labelled |  |  |
| 3 | Carts/buckets for transferring items from store clean |  |  |
| 4 | Closed waste bin |  |  |
| K | **PHARMACY** |  |  |
| 1 | Clean and dry and tidy (no visible dirt/litter-walls, floors, roof, fixtures, doors and other fixtures) & well ventilated |  |  |
| 2 | Closed waste bins for different wastes |  |  |
| 3 | Hand hygiene Materials (soap, water/ABHR) |  |  |
| 4 | Disposable paper towels available |  |  |
| **L** | **WASTE MANAGEMENT AREA** |  |  |
| 1 | Waste kept as segregated at final disposal |  |  |
| 2 | Incinerator functional |  |  |
| 3 | Hand washing facilities available (soap, water) |  |  |
| 4 | Hand wash basin clean |  |  |
| 5 | Staff put on PPE (gown/coverall, boots, heavy duty gloves, mask, googles/face shield) |  |  |
| **M** | **STAFF HEALTH** |  |  |
| 1 | Staff screened for COVID-19 symptoms |  |  |
| 2 | All staff wear clean uniform/scrubs |  |  |
| 3 | All staff wear closed shoes |  |  |
| 4 | All staff use PPE correctly |  |  |
| **N** | **Personal Protective Equipment (PPE)** |  |  |
| 1 | PPE supplies are stored off the floor and in a dry place |  |  |
| 2 | Daily stock taking done |  |  |
| 3 | Different PPE stored and labelled for easy identification |  |  |
| 4 | PPE stored in Clean and dry & well-ventilated room |  |  |

**Table 9: Bi-annual IPC supportive supervision checklist for general health facilities in Rohingya refugee camps in Cox’s Bazar.**

| Implementing Partner(s)____________Facility name and UID___________ Visiting team ________________Date of visit________________ | | | |
| --- | --- | --- | --- |
|  | | | |
|  | **Standard per area under observation** | **Findings** | **Suggested actions for improvement** |
| **A** | **SCREENING, WAITING AND TRIAGE AREA** |  |  |
| 1 | Screening tools functional (Registers, questionnaires, thermometer, stadiometer, weighing scale) |  |  |
| 2 | Clean and dry floor, walls and roof (no visible dirt/litter/wetness) & well ventilated |  |  |
| 3 | PPE Stock for HCW present (gown, mask, gloves, face shield, apron) |  |  |
| 4 | 1 m kept between patients, patients & HCW |  |  |
| 5 | Hand washing facilities available (soap, water/ ABHR, signage) and clean |  |  |
| 6 | Waste segregation bins (available, well labelled, visibly clean, liners, covers, < 3/4 full) |  |  |
| **B** | **EMERGENCY / OBSERVATION ROOM** |  |  |
| 1 | Floor, walls and roof Clean and dry (no visible dirt/litter/wetness) & well ventilated |  |  |
| 2 | The Examination bed is clean (no visible blood stains, clean linen) |  |  |
| 3 | Biomedical equipment (oxygen cylinder, concentrator, sucker, patient monitor, etc) are clean |  |  |
| 4 | All fixtures (spotlight, fans, chairs, hand wash basin etc) are clean (no visible dirt/litter/wetness) |  |  |
| 5 | Hand hygiene materials available (soap, water, tissue, signage) |  |  |
| 6 | Waste segregation bins (available, well labelled, clean, liners, covers, < 3/4 full) |  |  |
| **C** | **CONSULTATION ROOM(S) (GENERAL, NCD, EPI, NUTRITION)** |  |  |
| 1 | Clean and dry floor, walls and roof (no visible dirt/litter/wetness) & well ventilated |  |  |
| 2 | PPE Stock for HCW present (gown, mask, gloves, face shield, apron) |  |  |
| 3 | Hand washing facilities available (soap, water/ ABHR, signage) |  |  |
| 4 | Waste segregation bins (available, well labelled, visibly clean, liners, covers, < 3/4 full) |  |  |
| 5 | Examination bed is clean (no visible blood stains, clean linen) |  |  |
| 6 | Medical equipment clean and stored in closed container |  |  |
| 7 | All fixtures (Hand wash basin, lights, fans, chairs, cupboards, tables, etc) are clean |  |  |
| **D** | **PATIENT WARDS** |  |  |
| 1 | Clean, dry and tidy (floor, walls and roof) & well ventilated |  |  |
| 2 | Hand washing facilities available (soap, water, signage)/ABHR available at point of care |  |  |
| 3 | Fixtures (hand wash basin, Lights, switches, patients’ cupboards, IV stands, etc) clean |  |  |
| 4 | Beds, linen, screens, bed pans and other containers visibly clean |  |  |
| 5 | Waste segregation bins (available, well labelled, clean, liners, covers < 3/4 full) |  |  |
| 6 | Medication preparation area and nursing trolley are clean and well organized |  |  |
| 7 | Biomedical equipment visibly clean (glucometers, pulse oximeters, oxygen cylinders, concentrators) |  |  |
| 8 | Urinary drainage bags off floor |  |  |
| 9 | Date of insertion of IV catheters is registered |  |  |
| 10 | IV infusion sets are dated and discard (maximum) after 24 hours |  |  |
| 11 | Open ampoules are immediately discarded. Open vials are properly labelled (volume, date, time if MDV) and open syrup bottles are properly labelled (volume, date and time of expiry) |  |  |
| **E** | **ISOLATION ROOM (FLU CORNER, MATERNITY RED ZONE, AWD ISOLATION, ETC)** |  |  |
| 1 | Clean and dry floor, walls and roof (no visible dirt/litter/wetness) & well ventilated |  |  |
| 2 | PPE Stock for HCW present (gown, mask, gloves, face shield, apron, etc) |  |  |
| 3 | Hand washing facilities available (soap, water/ ABHR) |  |  |
| 4 | Waste segregation bins (available, well labelled, visibly clean, liners, covers, < 3/4 full) |  |  |
| 5 | Examination bed is clean (no visible blood stains, clean linen) |  |  |
| 6 | Medical equipment is clean and supplies and consumables stored in closed container |  |  |
| 7 | All fixtures and beds (hand wash basin lights, fans, chairs, etc) are clean (no visible dirt/wetness) |  |  |
| 8 | Different concentrations of chlorine prepared and labelled (mixing & discarding time recorded) |  |  |
| 9 | Separate donning and doffing areas (clean, waste segregated, signages, hand hygiene materials, well-lit and ventilated) |  |  |
| **F** | **LABORATORY** |  |  |
| 1 | The lab is visibly clean (roof, floor, walls), dry, no litter and ventilated |  |  |
| 2 | Hand wash station (water, soap and paper towel, signage) and ABHR is also available |  |  |
| 3 | Waste segregation bins (available, well labelled, visibly clean, liners, covers, < 3/4 full) |  |  |
| 4 | PPE is available (gloves, gowns, mask, respirators, face shield, etc) |  |  |
| 5 | All staff wear working clothes (scrubs, closed shoes) & don’t wear jewelry, rings or bracelets |  |  |
| 6 | Machines and other fixtures (lights, trays, worktables, machines and all fittings) are visibly clean |  |  |
| G | **ASRH, MHPSS, BREAST FEEDING ROOM** |  |  |
| 1 | Clean and dry floor, walls and roof (no visible dirt/litter/wetness) & well ventilated |  |  |
| 2 | 1 m kept between patients, patients & HCW |  |  |
| 3 | Hand washing facilities available (soap, water/ ABHR, signage) |  |  |
| 4 | Waste segregation bins (available, well labelled, visibly clean, liners, covers, < 3/4 full) |  |  |
| 5 | All fixtures (light, fans, chairs, hand wash basin, etc) are clean (no visible dirt/litter/wetness) |  |  |
| **H** | **STAFF RESTING AREA** |  |  |
| 1 | Clean, dry and tidy (floor, walls and roof)- no visible dirt/litter/wetness &well ventilated |  |  |
| 2 | Hand hygiene materials (water, soap/ABHR, Signages) |  |  |
| 3 | Furniture and other fixtures (hand wash basin, fans, TV, etc) clean |  |  |
| 4 | Waste segregation bins (available, well labelled, visibly clean, liners, covers, < 3/4 full) |  |  |
| 5 | Disposal paper towels/tissues |  |  |
| **I** | **KITCHEN/DINING** |  |  |
| 1 | Clean and dry (no visible dirt/litter/wetness) & well ventilated |  |  |
| 2 | Kitchen trolley, drinking water station, utensils, hand wash and kitchen sinks are clean and rust free |  |  |
| 3 | Cooking equipment clean (no visible dirt-in and outside) |  |  |
| 4 | Fridge and microwave clean (no visible dirt-in and outside) |  |  |
| 5 | Hand washing facilities available (soap, water) |  |  |
| 6 | Kitchen staff put on apron and head cover |  |  |
| 7 | Fire extinguisher available and functional (including signage on use) |  |  |
| 8 | Waste segregation bins (available, well labelled, visibly clean, liners, covers, < 3/4 full) |  |  |
| **J** | **TOILETS AND SHOWER ROOMS** |  |  |
| 1 | clean (no visible dirt/litter-walls, floors, roof, fixtures, bucket, water pot, doors, soap case) |  |  |
| 2 | Hand washing facilities (soap and water, signage) |  |  |
| 3 | Cleaning roster up to date and duly signed |  |  |
| 4 | Provision of menstrual hygiene kit in female washroom (for example; sanitary pads) |  |  |
| **K** | **DECONTAMINATION AREA** |  |  |
| 1 | Clean and dry and tidy (no visible dirt/litter-walls, floors, roof, fixtures, doors) & ventilated |  |  |
| 2 | Enough different concentrations of chlorine prepared and labelled (mixing and discarding time) |  |  |
| 3 | Staff put on PPE (gown, boots, heavy duty gloves, mask, googles/face shield, heavy duty apron) |  |  |
| 4 | Job aids (guides, SOPs, including timing for different procedures etc) for decontamination displayed and followed (linen, PPE, Equipment) |  |  |
| 5 | Uni directional flow of linen and equipment followed |  |  |
| 6 | All machines (washing, drying, autoclave) are clean (inside & outside) |  |  |
| 7 | washing sinks, basins, buckets, mops are clean, disinfected and stored dry |  |  |
| 8 | Hand washing facilities available (soap and water, signage) |  |  |
| **L** | **STORAGE AREA** |  |  |
| 1 | Clean and dry and tidy (no visible dirt/litter-walls, floors, roof, doors and other fixtures) &well ventilated |  |  |
| 2 | All items separately and neatly stored off the ground and labelled |  |  |
| 3 | Carts/buckets for transferring items from store clean |  |  |
| 4 | Closed waste bin |  |  |
| M | **PHARMACY/PHARMACY STORE** |  |  |
| 1 | Clean, dry and tidy (no visible dirt -walls, floors, roof, fixtures, and doors) & well ventilated |  |  |
| 2 | Temperature regulation working correctly |  |  |
| 3 | Hand hygiene Materials available (soap, water/ABHR) |  |  |
| 4 | Disposable paper towels available |  |  |
| 5 | Waste segregation bins (available, visibly clean, liners, covers, < 3/4 full) |  |  |
| **N** | **WASTE MANAGEMENT AREA** |  |  |
| 1 | Waste kept as segregated at final disposal (placenta pit, glass pit, sharps pit, ash pit, food) |  |  |
| 2 | Incinerator functional |  |  |
| 3 | Hand washing facilities available (soap, water) |  |  |
| 4 | Hand wash basin and other fixtures clean |  |  |
| 5 | Staff put on PPE (gown/coverall, boots, heavy duty gloves, mask, googles/face shield, heavy duty apron and if feasible have provision for doffing PPE) |  |  |
| **O** | **STAFF HEALTH & SAFETY** |  |  |
| 1 | All staff have been vaccinated against Hep-B |  |  |
| 2 | All staff in clean uniform/scrubs |  |  |
| 3 | All staff put on closed shoes during work |  |  |
| 4 | All staff use PPE correctly |  |  |
| 5 | Available reporting procedure for Accidental Exposure to Blood (AEB) and (complete PEP kits) |  |  |
| **P** | **PERSONAL PROTECTIVE EQUIPMENT (PPE) AND SUPPLIES** |  |  |
| 1 | Different PPE available for all activities (clinical and non-clinical activities) |  |  |
| 2 | IPC supplies are available (disinfectants, detergents, soap, cleaning materials,) |  |  |
| 3 | PPE and IPC supplies are stored off the floor, clean dry and ventilated room |  |  |
| 4 | Daily stock taking done and register maintained for all PPE and IPC supplies |  |  |
| 5 | Different PPE stored and labelled for easy identification |  |  |

**Table 10: Themes and subtheme analyzed.**

| **Theme** | **Subthemes** |
| --- | --- |
| **Theme 1**  **Implementation of IPC best practices** | IPC assessments for general HFs and SARI ITCs |
|  | COVID-19 IPC response plan |
|  | IPC Technical Working Group (TWG), leadership and coordination |
|  | Existence of adapted guidance documents |
|  | COVID-19 IPC trainings |
|  | Screening, early identification and isolation of suspected COVID-19 patients |
|  | Hand hygiene at strategic points in the HFs |
|  | IPC monitoring, audit and feedback in SARI ITCs |
|  | General masking for all patients |
|  | Health education of patients |
|  | IPC supportive supervision to SARI ITCs and HFs |
|  | Tracking of PPE and IPC supplies utilization in SARI ITCs |
|  | Design, infrastructure and use of Environmental controls in SARI and HFs |
|  | Health Care Waste management |
| **Theme 2**  **Challenges** | Frequent break down of incinerators |
|  | Limited PPE supply and irrational use |
|  | Inconsistent adherence to IPC practices by HWs |
|  | Limited investigation of HW infections and Healthcare-associated Infections (HAI) of COVID-19 |
|  | Lack of working uniform in many HFs |
|  | Lack of culture- and gender adapted work uniforms and PPE in SARI ITCs |
|  | Low implementation of IPC practices in the community |
| **Theme 3**  **Recommendations** | Establishment of an institutionalized IPC program for Cox’s Bazar district |
|  | Establishment of IPC monitoring, audit and feedback mechanisms in all HFs |
|  | IPC education and training |
|  | Strengthening public health and social measures in the communities |
